# Mapping medical careers: Questionnaire assessment of career preferences in medical school applicants and final-year students

**DOI:** 10.1186/1472-6920-4-18

**Published:** 2004-10-01

**Authors:** KV Petrides, IC McManus

**Affiliations:** 1 School of Psychology and Human Development Institute of Education University of London London WC1H 0AA, UK; 2 Department of Psychology University College London Gower Street London WC1E 6BT, UK

## Abstract

**Background:**

The medical specialities chosen by doctors for their careers play an important part in the workforce planning of health-care services. However, there is little theoretical understanding of how different medical specialities are perceived or how choices are made, despite there being much work in general on this topic in occupational psychology, which is influenced by Holland's RIASEC (Realistic-Investigative-Artistic-Social-Enterprising-Conventional) typology of careers, and Gottfredson's model of circumscription and compromise. In this study, we use three large-scale cohorts of medical students to produce maps of medical careers.

**Methods:**

Information on between 24 and 28 specialities was collected in three UK cohorts of medical students (1981, 1986 and 1991 entry), in applicants (1981 and 1986 cohorts, N = 1135 and 2032) or entrants (1991 cohort, N = 2973) and in final-year students (N = 330, 376, and 1437). Mapping used Individual Differences Scaling (INDSCAL) on sub-groups broken down by age and sex. The method was validated in a population sample using a full range of careers, and demonstrating that the RIASEC structure could be extracted.

**Results:**

Medical specialities in each cohort, at application and in the final-year, were well represented by a two-dimensional space. The representations showed a close similarity to Holland's RIASEC typology, with the main orthogonal dimensions appearing similar to Prediger's derived orthogonal dimensions of 'Things-People' and 'Data-Ideas'.

**Conclusions:**

There are close parallels between Holland's general typology of careers, and the structure we have found in medical careers. Medical specialities typical of Holland's six RIASEC categories are Surgery (Realistic), Hospital Medicine (Investigative), Psychiatry (Artistic), Public Health (Social), Administrative Medicine (Enterprising), and Laboratory Medicine (Conventional). The homology between medical careers and RIASEC may mean that the map can be used as the basis for understanding career choice, and for providing career counselling.

## Background

Medical careers begin as undifferentiated, and postgraduate training ends with most doctors specialised for a specific area of practice. Relatively little is known about the transition from the medical student, who can be seen as a relatively undifferentiated, totipotent 'stem doctor' [1,2], potentially capable of entering any speciality, through to the final, fully-differentiated specialist who is almost entirely restricted to one specialised area of medical work. Although medical career specialisation has been subject to a moderate amount of research (for reviews see e.g. [3,4]), some of it going back over half a century (e.g. [5]), much of that research has concentrated on the personal characteristics of individuals choosing particular careers (e.g. [6-8], on background factors in childhood influencing career choice (e.g. [8-10]), on associations with particular personality types (e.g. [11]), on the careers of specific groups, such as women doctors (e.g. [12]), on attitudes towards specific specialities, such as psychiatry (e.g. [13,14]) or anaesthetics (e.g. [15,16]), or has concentrated on the basic statistics necessary for workforce planning (e.g. [17,18]). There is, however, a lack of any broad theoretical framework in which to place career choice and specialisation.

UK medical education requires undergraduates to study a wide range of medical specialities, and most students will have sampled many of the broad areas of practice by the time they qualify. As a result, it is often assumed that students do not make their career choices until after they have finished at medical school, remaining agnostic about their final speciality choice until that time. However, not only medical school entrants (e.g. [19]), but even medical school applicants, a year or so earlier, at the typical age of about seventeen, often have surprisingly strong preferences for, and particularly, against, some medical careers (e.g. [20]). There is strong evidence, therefore, that career choice can be determined during or even before medical school ([21,22])). Thus, it makes sense to try and understand those preferences, which probably underpin eventual career choice.

Much research into medical careers does not take into account the broader research literature on non-medical careers (see [23-25]), or on socio-psychological models of the theoretical underpinnings of career choice (e.g. [26], [27], [28,29]). Consequently, medical careers research often fails to provide any broader theoretical framework or conceptualisation within which the empirical findings may be explained or which allow generalisations beyond the immediate data collected in the study (although there are exceptions, e.g. [30,31]).

The present study takes its origins in three separate sets of theoretical approaches, each of which examines different aspects of careers. None of these approaches, however, concerns medical careers specifically. Neither are they restricted to career choice in adulthood. Furthermore, at least one of them is specifically developmental, emphasising the processes by which career choice occurs and changes. The best place to begin this brief theoretical review is with the work of Gottfredson [27], who identifies the distinct processes of *circumscription *and *compromise *in career choice.

Careers differ in their demands, requiring different amounts of intellectual ability, manual skill, long-term commitment, or willingness to work in particular environments, and can be better suited to particular personalities, aptitudes, and physical dispositions. Individuals also differ, having different aptitudes, interests and abilities. Career choice therefore involves people considering the entire range of careers and then circumscribing those which they regard as broadly acceptable, making their eventual choices within that subset.

An important practical point highlighted by studies such as Gottfredson's is that choices tend to be *negative*, meaning that careers are rejected because they do *not *have attributes which are consonant with the person making the choice, rather than positively chosen for their special suitability.

Once circumscription has taken place, a number of possible careers still remain. The second stage of choice is *compromise*. Because of various practical constraints, certain careers are restricted in the number of people they can accommodate or they are unsuitable in other terms, such as their geographical location or the remuneration they can provide. The eventual career chosen is one that 'satisfices,' [32] being realistically good, though not optimal. The applications of this theory to medical careers are self-evident and describe many of the problems facing medical students and junior doctors.

Implicit in Gottfredson's conceptualisation is the concept of a *map *of careers. In her 1981 paper she provides an example a two-dimensional representation of 129 occupations which have been scored in terms of 'Prestige level' (high *vs *low) and 'sextype rating' (masculine *vs *feminine). When careers are mapped into this space, the process of circumscription involves drawing an area within which careers are acceptable to a person, being neither too masculine nor too feminine, nor being too high in terms of their prestige and hence effort required, nor too low, and hence insufficiently rewarding. A primary concern of the present study is the nature of the map underlying medical careers, and on which circumscription eventually takes place.

**Figure 1:**
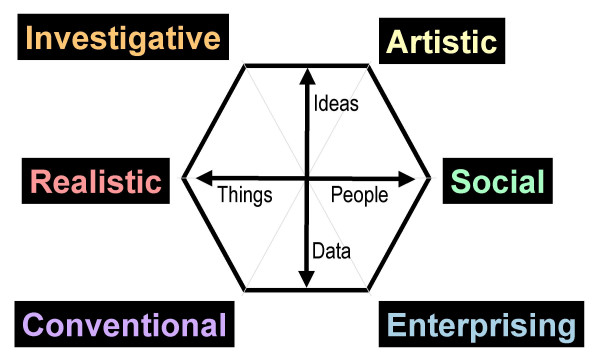
The hexagon of Holland's RIASEC typology, along with the Things-People and Ideas-Data dimensions proposed by Prediger (1982).

Perhaps the most influential study of the structure of career preferences is that of Holland [26], an overview and critical analysis of which can be found in the special issue of the *Journal of Vocational Behavior *published in 2000 (e.g. [25]; see also [33] and [34]). Holland's theory suggests that careers can be organised into six broad types, which can be represented around a hexagon (see figure 1), and which are often known by the acronym RIASEC, standing for Realistic, Investigative, Artistic, Social, Enterprising and Conventional. In Holland's original conceptualisation the specific orientation of the hexagon is arbitrary to rotation, but subsequent analyses have suggested that the hexagonal structure can be reduced to two dimensions [35,36]. One dimension runs from Realistic to Social, involving careers that are primarily Things-oriented rather than People-oriented. The second orthogonal dimension runs from midway between Enterprising and Conventional to midway between Artistic and Investigative, and involves careers varying from those that are primarily Data-oriented to those that are primarily Ideas-oriented. Holland's RIASEC model provides an appropriate two-dimensional space in which Gottfredson's circumscription model can apply [37].

Although Holland's work suggests how careers might be mapped, and Gottfredson's work suggests how career choices might take place within the space underlying those careers, a missing link in the overall picture concerns how individuals choose within the space. This is a significant question because individuals are expected to circumscribe in different ways according to their particular personalities and abilities. Ackerman [28,29] has described how intellectual ability and personality relate to Holland's RIASEC model. Measures of intellectual ability primarily correlate with interest in the Realistic, Investigative and Artistic careers, people with higher verbal abilities preferring careers in Artistic and Investigative careers, and people with higher spatial and mathematical abilities preferring Realistic and Investigative careers. In contrast, measures of personality mainly correlate with the SEC components of RIASEC. Ackerman uses the Big Five typology of personality (see [38], [39]), and shows that Extraversion primarily correlates with an interest in Social and Enterprising careers, whereas Conscientiousness correlates with an interest in Conventional and Enterprising careers. The personality dimension of Openness to Experience is to some extent a hybrid between intellectual ability and personality, and tends to correlate positively with Artistic, Investigative and Realistic careers, and negatively with Conventional careers. This pattern is similar to that which Zhang has reported in which the RIA cluster of careers relates to a deep approach to learning [40,41], whereas the SEC cluster relates to a strategic approach to learning [42].

Between them, the models of Holland, Ackerman and Gottfredson provide, respectively, a good conceptualisation of i) the structure of careers and career preferences, ii) the correlations of careers with ability and personality, and iii) the developmental processes by which career choices are made. The question for medical education is the extent to which these approaches are appropriate for understanding medical career choice. If they are valid, then that will allow the much broader research literature from career choice in general to inform the more specific area of medical career choice. Underpinning the models of both Ackerman and Gottfredson is Holland's picture of a relatively simple, two-dimensional career map, onto which ability and personality can project, and on the basis of which career choices can develop. We therefore have two main objectives in this paper; firstly, to use data on career preferences from three separate cohorts of medical students, both at the time of application and in their final year at medical school, in order to derive a map of medical careers. And second, to assess the extent to which this specific map of medical careers is homologous to Holland's more general map of a broad range of careers.

The data collected in our studies consist of ratings of attractiveness of different medical careers on a five-point scale, ranging from 'Definite intention to go into this' through to 'Definite intention not to go into this'. However, our primary interest for the purpose of deriving a map of careers is not in *career preference*, but rather in *career similarity. *If a student has a preference for career A and career B, but has no interest in career C and D, it follows that career A is probably relatively close to career B on the map, and career C is relatively close to career D, whereas careers A and B are likely to be more distant from careers C and D. A matrix of similarities between all possible pairs of a large number of careers from a large number of students then allows one to construct the underlying map (just as, in a classic example, a knowledge of the geographical closeness, or the drive-time, between many pairs of towns in a country allows one to reconstruct a map of the country [43,44]). The statistical technique is known as multi-dimensional scaling (MDS).

Although conventional MDS can reconstruct the underlying map showing the relations between a number of objects, the map itself is *arbitrary to rotation. *Turning the map through any angle does not change any of the distances between pairs of objects, and therefore the axes of the map cannot be known – in the case of a geographical map, there is no indication of the north-south and east-west axes. The problem of the arbitrariness of dimensions can be circumvented by means of a variant of MDS known as INDSCAL (Individual Differences Scaling) [44,45]. This method analyses the similarity matrices either of individual subjects or of groups of subjects who are likely to differ, so that, for instance, one might have groups based on sex and age, the presumption being that older students may have different career preferences from their younger peers, and female students may have different preferences from their male peers. INDSCAL then allows the assignment of axes, it being likely that the grouping variables will mainly affect one rather than all of the dimensions on which the map is represented. An example in the case of geographical distance might be to examine the time of travel between pairs of towns in winter and summer. Inclement winter weather will increase the time of travel in the more northerly towns, but the dimension of east-west will have little impact on the measures. In this paper, we use INDSCAL to construct our maps of medical careers, so that the axes are identified and not arbitrary to rotation.

## Methods

Two different types of data have been used in the present study. The bulk of the analysis looks at data collected during studies of medical student selection and training, and can be used to map medical careers. A subsidiary, but important, analysis looks at a large convenience sample of people of different ages who were taking part in a survey about careers in general, and were asked about their interest in a range of careers, of which only a few were medical. These latter data allowed us both to calibrate specific medical careers in the context of the general Holland typology, and to validate the INDSCAL methodology for deriving a map of careers.

### Medical student data

The data were collected during three longitudinal studies of medical student selection, the first of which began in the autumn of 1980, looking at students who had applied for entry to medical school in 1981 [46,47], the second began in the autumn of 1985, studying applicants for entry to medical school in 1986 [48,49], and the third began in 1990, studying applicants for entry to medical school in 1991 [50,51]. The 1981 and 1986 cohort studies were restricted to students applying for entry to St. Mary's Hospital Medical School in London, although since applicants had each applied to five or six medical schools, many students entered schools other than St. Mary's. The 1991 cohort study looked at applicants to five different English medical schools, and because each applicant applied to several schools, these applicants represented 70% of all applicants and entrants to UK medical schools in that year. In each survey, applicants were sent questionnaires as soon as possible after UCCA, the central universities admission system, had received their application and entered their names had been onto the computer database. In general this was many weeks or even months before applicants were asked to come for interview, or were sent decisions on whether they had been accepted or rejected. The data are therefore to a great extent properly prospective.

For the present paper, the analysis of the 1981 and 1986 cohort data considers data on all applicants who replied to our questionnaires, whereas the 1991 cohort, which was very much larger, considers only those questionnaire respondents who entered medical school (although we will generally refer to this group as 'applicants' since that reflects the time at which the questionnaire was completed). In additional file 1 we present separate information for the entire 1991 cohort which shows that there are unlikely to be response biasses, either due to differences between accepted and rejected applicants, or due to not all entrants responding to the final-year questionnaire. Response rates in the 1981, 1986 and 1991 applicants surveys were 85%, 93% and 93% [46,48,50].

Students who entered medical schools in 1981, 1986 or 1991 (or in a few cases due to deferred or repeated entry, in 1982, 1987 or 1992) were followed up as final-year students in 1986 (or 1987), in 1991 (or 1992) and 1996 (or 1997). Students still in medical school were identified through their medical schools, and questionnaires sent to those medical schools. Response rates were 65%, 50%, and 56% in the follow-up of the 1981, 1986 and 1996 cohorts of students in their final year [49,51].

The questionnaires used in the study, both at application and in the final year, were detailed, typically covering 16 sides of A4, and the results reported here concern only one of the questions asked. Career preferences were assessed by a question which used the rubric,

"Below is a detailed list of specialities in which a medical career can be pursued. Please indicate your attitude towards each speciality as a possible career. If you either know nothing about a speciality, or have no opinions about it at all, simply leave that answer blank".

A list of specialities followed, each of which was rated on a five-point scale, for which the categories were, "Definite intention to go into this", "Very attractive", "Moderately attractive", "Not very attractive", and "Definite intention *not *to go into this".

The list of specialities varied a little over the different surveys, becoming slightly more extensive as the years passed. The original list was based on the questionnaire distributed as part of the Royal Commission on Medical Education of 1968 [52] (The Todd Report). The questionnaire for 1981 applicants had 24 questions. The final-year questionnaire for the 1981 applicants had 26 questions, the two new categories being "Pre-clinical teaching" and "Geriatric Medicine". The questionnaire for the 1986 applicants was the same as that for the 1981 applicants except that it had 25 specialities, "Geriatric medicine" having been added. The final-year questionnaire for the 1986 cohort had 27 questions, the 25 used for applicants, with the addition of "Genito-Urinary Medicine" and "Infectious Diseases". The applicant questionnaire for the 1991 cohort had the same 27 questions as did the final-year questionnaire for the 1986 cohort. The final-year questionnaire for the 1991 cohort was similar to that for the applicants except that it had 28 questions, "Radiology/Radiotherapy" having been split into two separate specialities. In the present study all of the questionnaires have been used in the form in which they were originally administered, the only omission being the speciality "Pre-clinical teaching", which was used in one survey only and is of little interest.

### The general population sample

This questionnaire was completed by a sample of 1026 subjects, stratified by age using a median split (≥ 42; <42) and by sex. It asked about the suitability of twenty-four different careers for the person. Twenty careers were derived, as far as possible, from Holland's RIASEC classification, with at least three in each of the six categories. In addition there were four categories which were medical (Anaesthetist, Hospital Doctor, Psychiatrist and Surgeon). The rubric was,

"Below is a list of careers. Please indicate for each one how much you think it might have been suitable for you as a career".

Each career was rated on a five-point scale, ranging from 'Extremely suitable', through 'Very suitable' and 'Quite suitable', to 'Not very suitable' and 'Completely unsuitable'. The subjects were a convenience sample obtained from amongst friends and relations by a first year lab class at University College London, each student being responsible for obtaining a group of twelve subjects, stratified by age and sex.

### Statistical analysis

INDSCAL analysis was carried out using the ALSCAL program within SPSS 10.1. Data in each subset were broken down into four groups by age and sex, age referring to mature vs non-mature students in the medical student samples (≤ 21; >21) and to subjects aged <42 or ≥ 42 in the general population sample.

The raw data, which were collected on a 5 point Likert scale, were transformed into Euclideanbased dissimilarities for all combinations of career pairs, using the PROXIMITIES program in SPSS. Four different dissimilarity matrices were produced (nonmature males, nonmature females, mature males, and mature females), and these matrices provided the basis for the INDSCAL analysis that involved minimisation, in Euclidean space, of the discrepancies between the career dissimilarities and the corresponding interpoint distances on the map. The loadings of each career on the two extracted dimensions were then plotted onto the figures to provide the maps.

The dimensionality of MDS/INDSCAL analyses can be assessed, in a manner analogous to that used in factor analysis in which eigenvalues are plotted against components. In MDS one plots a measure of 'stress' (in effect, the opposite of goodness-of-fit) against the number of dimensions which have been extracted. If too few dimensions have been extracted then the stress is high, the model not accounting adequately for the richness of the data. The optimal number of dimensions is typically indicated by a sudden 'dog-leg' in the stress plot.

## Results

We consider firstly the general population sample since it both validates the method which we will subsequently use for the medical student samples, and also helps calibrate the axes.

### The general population sample

**Figure 2:**
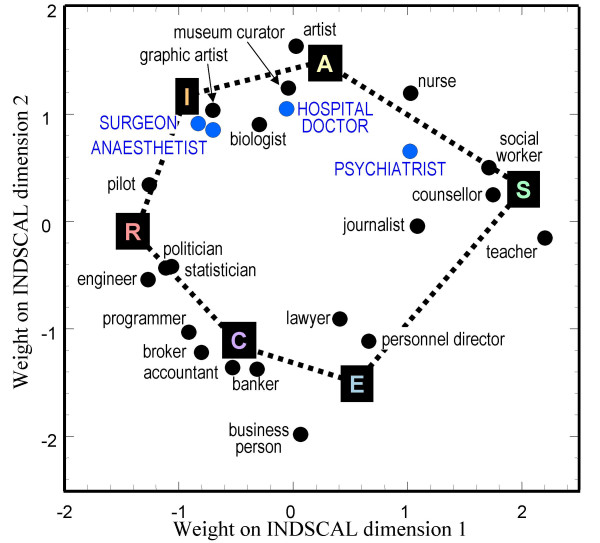
INDSCAL group space of the career preferences expressed by the general population sample. The locations of the labels R, I, A, S, E and C are approximate and are only for guidance and orientation. The four medical specialities are shown in blue so that they are more visible.

The questionnaire was completed by 1044 subjects, 49.2% of whom were male, and 46% aged over 42. Multidimensional scaling used the INDSCAL method, with the four groups comprising older and younger males and older and younger females. The stress plot indicated that there were two major underlying dimensions in the data, as Holland's typology would suggest (see also Prediger [53,35]). The locations of the different careers are shown in figure 2. There is good evidence for Holland's RIASEC typology, and the letters R, I, A, S, E and C have been placed on the graph to clarify interpretation. Pilot and Engineer are typical of Realistic careers, Biologist of Investigative careers, Artist and Museum Curator of Artistic careers, Social Worker, Counsellor and Teacher of Social careers, Personnel Director and Lawyer of Enterprising careers, and Accountant and Computer Programmer of Conventional careers. For this non-medical group of subjects, the four medical careers are all placed in the top half of the figure, with surgeon and anaesthetist closest to Investigative, and Psychiatrist closest to Social.

Of some importance, given the arbitrariness of the Holland hexagon to rotation in conventional MDS, is that the INDSCAL analysis clearly sets one axis as running from R to S, with the other axis orthogonal to that, running from I and A to C and E. These are similar to the Things-People and Ideas-Data dimensions shown in figure 1.

### The medical student samples

Sample sizes for the medical student studies were 1135, 2032 and 2973 for the students in the 1981, 1986 and 1991 cohorts (and these samples consisted of all *applicants *in the 1981 and 1986 cohorts, and all *entrants *in the 1991 cohort), and were 330, 376 and 1437 for the final-year students in the 1981, 1986 and 1991 cohort studies. The INDSCAL analyses were restricted to those subjects for whom complete career information was available; this consisted of 538 applicants and 312 final-year students in the 1981 cohort, 1118 applicants and 301 final-year students in the 1986 cohort, and 1638 entrants and 1437 final-year students in the 1991 cohort. See additional file 1 for details of the breakdown of samples by sex and maturity.

The dimensionality of the medical student samples was assessed by carrying out a standard multi-dimensional scaling analysis (i.e. MDS, not INDSCAL), separately for the combined applicant data and the combined final-year data from the three cohorts. The stress formula attempts to quantify the discrepancies between the fitted distances in the model and the observed dissimilarities among the career ratings, with larger values indicating poorer fit. It is obviously the case that the more dimensions are extracted, the better the fit of the model and, hence, the lower the stress value. However, it is also the case that a greater number of dimensions complicates interpretation and may lead to overfitted and unstable solutions. The stress levels with 1,2,3,4,5, and 6 dimensions were .352, .174, .112, .077, .059 and .048 for applicants, and .390, .174, .112, .082, .064 and .052 for final-year students. For the final-year students, it is clear that two dimensions are necessary, and that there is little advantage of adding extra dimensions. The applicant data are slightly less clear and although there is still no doubt that at least two dimensions are necessary there is a suggestion that a third dimension may be of value. Subsequent scrutiny of models with three dimensions suggested that the third dimension was contributed almost entirely by one or two specialities such as forensic medicine, which have a high public and media profile, but which form only a small proportion of medical personnel. It was, therefore, felt to be safe to extract two dimensions, particularly since Holland's typology provided an *a priori *expectation that there would be two dimensions.

#### INDSCAL analyses

Separate analyses were carried out for the applicant and final-year data in each of the three cohorts. In each case, data were broken down into sub-groups according to sex (male-female) and age (mature at entry to medical school, *i.e. *>21 yrs old; or typical post-school entry, at ≤ 21 years old). INDSCAL analyses can clarify the underlying dimensions within data as long as the sub-groups are likely to vary along those dimensions. It should be noted that many studies have found sex differences in medical career interest (e.g. [54]), and younger students are also likely to have different attitudes towards careers than their non-mature counter-parts [55].

**Figure 3:**
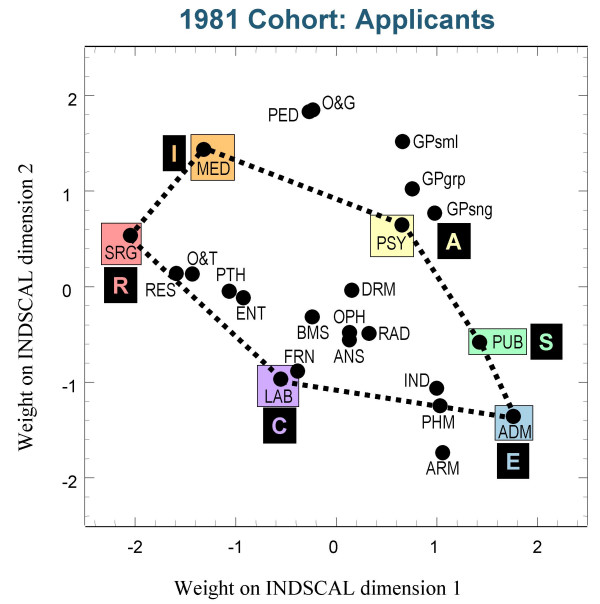
The INDSCAL group space for the medical specialities for the applicants in the 1981 cohort. For abbreviations see the Abbreviations section.

**Figure 4:**
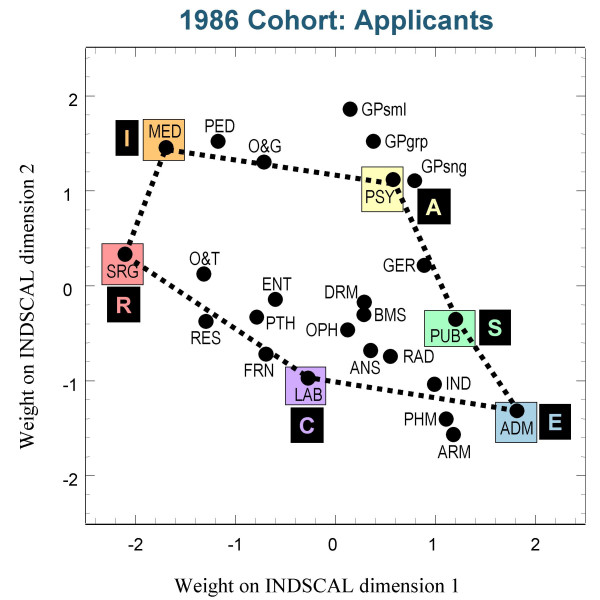
The INDSCAL group space for the medical specialities for the applicants in the 1986 cohort. For abbreviations see the Abbreviations section.

**Figure 5:**
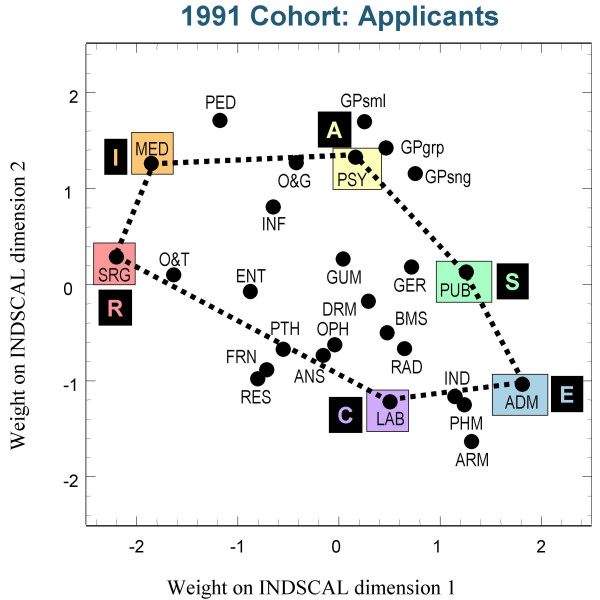
The INDSCAL group space for the medical specialities for the entrants in the 1991 cohort. For abbreviations see the Abbreviations section.

**Figure 6:**
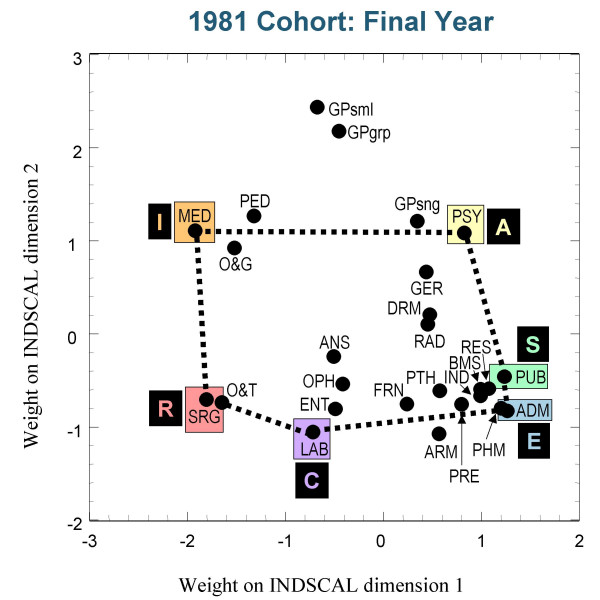
The INDSCAL group space for the medical specialities for the final-year medical students in the 1981 cohort. For abbreviations see the Abbreviations section.

**Figure 7:**
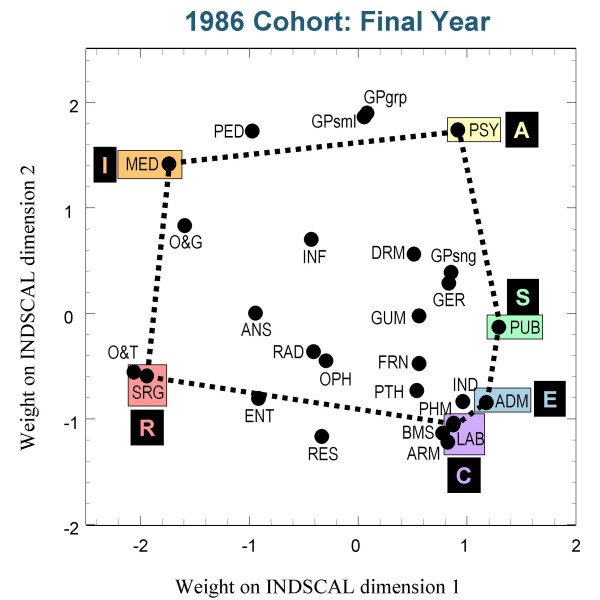
The INDSCAL group space for the medical specialities for the final-year medical students in the 1986 cohort. For abbreviations see the Abbreviations section.

**Figure 8:**
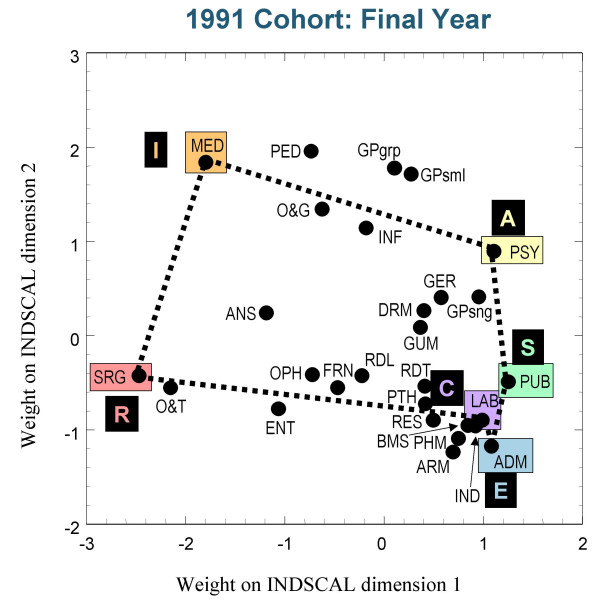
The INDSCAL group space for the medical specialities for the final-year medical students in the 1991 cohort. For abbreviations see the Abbreviations section.

Figures 3,4 and 5 show the group plots of the different specialities in applicants to medical school, and figures 6, 7 and 8 show the group plots for the specialities in final-year medical students. In order to help interpret these plots, and for reasons which will become clearer later, we have joined together the data points for Surgery, Hospital Medicine, Psychiatry, Public Health, Administrative Medicine and Laboratory Medicine. For the applicants, it is now clear that these specialities are arranged approximately in the form of a hexagon, with Surgery at the extreme left and Administrative Medicine at the bottom right-hand corner. The pattern shown in the final-year students is similar, Surgery still being at the left-hand side, and Administrative Medicine at the bottom right. Although there are some minor differences between the three cohorts, the broad picture is of overall similarity in the structure of the maps.

**Figure 9:**
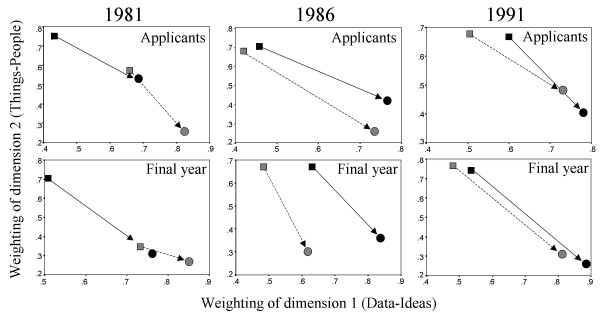
INDSCAL source spaces for the applicants/entrants and final-year medical students in the 1981, 1986 and 1991 cohorts. Square symbols are for male subjects and circles for female subjects. Solid symbols are for younger students, whereas hatched symbols are for mature students. To help visualisation, the solid arrows connect from younger males to younger females, whereas dashed arrows connect from mature males to mature females. See text for further details of interpretation.

The maps shown in figures 3 to 8 are, in INDSCAL terminology, group spaces [44,45]. They are, however, composed of several different sources, broken down by age and sex. Maps can also be produced of 'source space' which shows how the groups differ in their relative weighting of the two extracted dimensions. Figure 9 shows the source spaces for the applicant and final-year student data in the three cohorts. The vertical axis represents the relative importance of the Things-People dimension, whereas the horizontal dimension shows the importance of the Data-Ideas dimension. It should be noted that these axes do *not *mean that, say, People are more important than Things, but that the Things-People dimension is more differentiated than the Data-Ideas dimension (just as, say, in a map of Italy or Chile, there is far more north-south differentiation than east-west, as they are long-thin countries). In each of the six analyses, the male subjects put more emphasis on the Things-People dimension whereas the female subjects put more emphasis upon the Data-Ideas dimension (and hence the male subjects tend to be in the top left corner and the female subjects in the bottom-right). In the 1981 cohort there is also a suggestion that younger subjects put more emphasis on the Things-People dimension, and older subjects on the Data-Ideas dimension for differentiating careers, but the effect is smaller in the 1986 cohort, and barely visible in the 1991 cohort, suggesting a possible change in the way these groups perceive medical careers. In interpreting these analyses it should be noted that although the absolute size of the various groups was more than adequate for the INDSCAL analyses, the group weights for the mature candidates (male and female) in the 1981 and 1986 finalyear data were based on very small samples, ranging between 5 and 17 participants, and may, therefore, be somewhat unstable.

## Discussion

The primary objectives of this study were to use the empirical method of individual differences scaling to derive maps of the underlying perceived structure of medical career specialities, and to assess the extent to which those maps are similar to those described by Holland in his hexagonal representation of the RIASEC groups of careers. That this method is a valid way of deriving Holland's structure in general is seen in figure 2, in which a broad range of non-medical careers is assessed by non-medical individuals, and the RIASEC structure is readily derived. Of particular importance is that because the analysis used INDSCAL, the dimensions are not arbitrary to rotation, and that the R-S dimension (corresponding to the Things-People dimension) and the IA-EC dimension (corresponding to the Ideas-Data dimension) are the basic underlying structure, as shown by Prediger [35,53]. The "Things-People" dimension also bears a strong similarity to the Technique orientation and People orientation which has also been described in relation to medical specialities [56].

The general population sample also rated four medical specialities, with Surgery and Anaesthetics at one extreme, and Psychiatry at the other, and these medical specialities differed principally along the R-S dimension. That Surgery and Anaesthetics are more concerned with Things, and Psychiatry is more concerned with People fits well with the reduction of Holland's hexagon to the two dimensions of Things-People and Ideas-Data. It is also worth noting that *all *of the four medical specialities are seen by the general public as being primarily concerned with Ideas rather than with Data, as surely befits medical careers.

The MDS analyses demonstrate that the representation of the various medical specialities by the medical student samples can be captured within a two-dimensional space, as Holland had suggested. The maps shown in figures 3 to 8 indicate that the structures are broadly similar across the three cohorts, and that although there are some minor differences between the applicants and the final-year students, it is the case that overall the similarities are more impressive than the differences. The crucial question therefore concerns whether the medical student maps are homologous to those of Holland's RIASEC typology. If there is a homology, then one may ask what are the Realistic, Investigative, Artistic, Social, Enterprising and Conventional specialities of medicine.

From scrutinising figures 3 to 8 we suggest that the RIASEC structure of medicine is typified by the six prototypical specialities of Surgery, Hospital Medicine, Psychiatry, Public Health, Administrative Medicine and Laboratory Medicine. It should be emphasised that in suggesting this we are not implying a direct comparability in the posts, rather a formal similarity within the limits imposed by being within the domain of medicine, as opposed to that of careers in general.

### Surgery – *Realistic*

Surgeons can be seen as the engineers of medicine, solving problems at high levels of mechanical and technical proficiency, with an emphasis upon practical skills, craftsmanship, and immediate and effective results.

### Hospital Medicine – *Investigative*

The core of Hospital Medicine (Internal Medicine) is diagnosis, achieved by carrying out appropriate investigations. Physicians typify the model of the 'scientist-practitioner', investigating symptoms and signs and relating them to the underlying pathophysiology of the patient.

### Psychiatry – *Artistic*

Psychiatrists, and also General Practitioners, have a more artistic approach to medicine, seeing, interpreting and responding imaginatively to a range of medical, social, ethical and other problems. The emphasis in many ways is on the uniqueness of the patient, the ideas that they are expressing, and the psycho-social theories and concepts which are necessary for interpreting the individual.

### Public Health – *Social*

Although most medicine is concerned with individual patients, the remit of Public Health is primarily social in the sense of applying medicine to society as a whole, treating the 'body politic'. It is noteworthy that in the maps, Public Health is not only at the Social end, but also closer to Data than to Ideas. Public Health manages social and community health by the appropriate analysis of data.

### Administrative medicine – *Enterprising*

The management of hospitals and health-care requires the creative skills of the business executive, the lawyer and the personnel director to achieve a smoothly running system. People, both patients and carers, are at the heart of any health-care system, and therefore administrative medicine is at the People end of the dimension.

### Laboratory Medicine – *Conventional*

The running of efficient systems in haematology, histopathology or chemical pathology requires many of the attributes shared with the accountant or the banker, including the willingness to develop, implement and follow standard procedures within a complex system. The emphasis is inevitably upon the things that do the measurements, and upon the data collected, rather than the ideas or people behind the data and the technology.

The analyses in this paper suggest that in our groups of students there is a broad similarity between preferences for medical careers and the typology found by Holland in careers in general, suggesting that the structures are homologous. Although our study has been restricted to medical students in the UK, our findings are likely to be generalisable, given that the patterns are found in three separate cohorts studied over a decade, and across medical school applicants and final-year students. Just as Holland's typology is found in most studies of careers, over a period of three decades and in many countries, despite a wide range of changes in society, in education, and in the nature of jobs and careers themselves, so we would predict that our typology of medical careers will be robust to such changes. To put it more strongly, we would predict that despite enormous changes in every aspect of medicine over two and a half millennia, just as Hippocrates recognised that surgery is different in many ways from other branches of medicine, and that not every doctor wishes or is able to be a surgeon, so the same applies today and will probably continue to apply as long as medicine is practised. That is likely to be so primarily, as Ackerman has suggested, because Holland's typology is underpinned by wide-ranging, broadly defined individual differences in aptitude and personality [36] which are also likely to be stable across time and cultures [57].

It may at first be felt that our approach to mapping careers is fundamentally different to that of Gale and Grant [58,59], who describe a questionnaire, the Sci-45, which has twelve sub-scales and allows discrimination between 45 different medical specialities as possible careers. However, the purposes of that instrument and our analyses are very different. Gale and Grant aimed at developing a practical instrument for counselling individuals, which would allow a detailed differentiation between careers. In contrast, we aimed at investigating and mapping the broad picture underlying careers. To use an analogy with geography, our map is primarily a large-scale representation of a region such as Britain, which lays out the main north-south and east-west axes and defines the broad regions of that map (Scotland, Wales, South of England, East Anglia), as well as placing the main cities, which are analogous to the specific careers. Gale and Grant in contrast are developing a method of differentiating between the various cities, particularly when, as say in the West Midlands conurbation, some cluster closely together within the map. We therefore expect that underlying the Gale and Grant questionnaire will be two broad dimensions equivalent to those which we have described.

## Abbreviations

INDSCAL Individual differences scaling

MDS Multidimensional scaling

RIASEC Realistic-Investigative-Artistic-Social-Enterprising-Conventional

Speciality abbreviations in figures 3 to 8.

ADM Administrative Medicine

ANS Anaesthetics

ARM Armed Forces

BMS Basic Medical Sciences

DRM Dermatology

ENT Ear, Nose & Throat

FRN Forensic

GER Geriatrics

GPlrg GP Large Group practice

GPsml GP Small practice

GPsng GP Single handed

GUM Genito-urinary medicine

IND Industrial Medicine

INF Infectious diseases

LAB Laboratory (Haematology, Clinical Chemistry, etc.)

MED Internal Medicine

O&G Obstetrics & Gynaecology

O&T Orthopaedics & Trauma

OPH Ophthalmology

PED Paediatrics

PHM Pharmaceutical Medicine

PSY Psychiatry

PTH Pathology

PUB Public Health

RAD Radiology/ Radiotherapy

RDL Radiology

RDT Radiotherapy

RES Research

SRG Surgery

## Competing interests

The authors declare that they have no competing interests.

## Authors' contributions

ICM had collected the data in the various surveys over a number of years. ICM and KVP jointly decided how to do the statistical analysis, and KVP was responsible for the programming and data analysis. ICM wrote the first draft of the paper, which was revised by KVP, with both authors being responsible for the final draft.

## Pre-publication history

The pre-publication history for this paper can be accessed here:



## Supplementary Material

Additional File 1 Additional analyses of data Click here for file

## References

[B1] Chant ADB (1991). Designing a doctor. Lancet.

[B2] Chant A (1989). The stem doctor.

[B3] Davis WK, Bouhuijs PA, Dauphinee WD, McAvoy PA, Alexander DA, Coles C (1990). Medical career choice: current status of research literature. Teaching and Learning in Medicine.

[B4] Dohn H (1996). Choices of careers in medicine: some theoretical and methodological issues. Medical Education.

[B5] Strong EK, Tucker AC (1952). The use of vocational interest scales in planning a medical career. Psychological Monographs.

[B6] Schumacher C (1964). Personal characteristics of students choosing different types of medical careers. Journal of Medical Education.

[B7] Kritzer H, Zimet C (1967). A retrospective view of medical speciality choice. Journal of Medical Education.

[B8] Monk MA, Thomas CB (1973). Personal and social factors related to medical specialty practice. Johns Hopkins Med J.

[B9] Paris J, Frank H (1983). Psychological determinants of a medical career. Can J Psychiatry.

[B10] Crimlisk H, McManus IC (1987). The effect of personal illness experience on career preference in medical students. Medical Education.

[B11] Bartnick L, Kappelman M, Berger J, Sigman B (1985). The value of the California Psychological Inventory in predicting medical students' career choice. Medical Education.

[B12] Stanley GR, Last JM (1968). Careers of young medical women. British Journal of Medical Education.

[B13] Walton HJ (1969). Personality correlates of a career interest in psychiatry. Br J Psychiatry.

[B14] Zimet CN (1975). Psychiatric specialty choice among medical students. Journal of Clinical Psychology.

[B15] Reeve PE (1980). Personality characteristics of a sample of anaesthetists. Anaesthesia.

[B16] Parkhouse J, Ellin DJ (1990). Anaesthetics: career choices and experiences. Medical Education.

[B17] Parkhouse J (1991). Doctors' careers.

[B18] Lambert T, Goldacre M, Parkhouse J (1998). Doctors who qualified in the UK between 1974 and 1993:age, gender, nationality, marital status and family formation. Medical Education.

[B19] Carline J, Cullen T, Dohner C, Schwarz R, Zinser E (1980). Career preferences of first and second year medical students. Journal of Medical Education.

[B20] McManus IC, Lefford F, Furnham AF, Shahidi S, Pincus T (1996). Career preference and personality differences in medical school applicants. Psychology, Health and Medicine.

[B21] Hutt R, Parsons D, Pearson R (1981). The timing of and reasons for doctors' career decisions. Health Trends.

[B22] Zeldow PB, Preston RC, Daugherty SR (1992). The decision to enter a medical speciality: timing and stability. Medical Education.

[B23] McManus IC (1997). Medical careers: Stories of a life. Medical Education.

[B24] Arthur MB, Hall DT, Lawrence BS, [Editors] (1989). Handbook of career theory.

[B25] Prediger DJ (2000). Holland's hexagon is alive and well – though somewhat out of shape: Response to Tinsley. Journal of Vocational Behavior.

[B26] Holland JL (1973). Making vocational choices: A theory of careers.

[B27] Gottfredson LS (1981). Circumscription and compromise: A developmental theory of occupational aspirations. Journal of Counseling Psychology Monograph.

[B28] Ackerman PL (1996). A theory of adult intellectual development: Process, personality, interests and knowledge. Intelligence.

[B29] Ackerman PL, Heggestad ED (1997). Intelligence, personality, and interests: Evidence for overlapping traits. Psychological Bulletin.

[B30] Mitchell WD (1975). Medical student career choice: a conceptualization. Social Science and Medicine.

[B31] Zimet C, Held M (1975). The development of views of specialities during four years of medical school. Journal of Medical Education.

[B32] Schwartz B (2004). The tyranny of choice. Sci Am.

[B33] Gati I (1991). The structure of vocational interests. Psychological Bulletin.

[B34] Gottfredson GD (1999). John L Holland's contributions to vocational psychology: A review and evaluation. Journal of Vocational Behavior.

[B35] Prediger DJ (1992). Locating occupations on Holland's Hexagon: Beyond RIASEC. Journal of Vocational Behavior.

[B36] Lippa R (1998). Gender-related individual differences and the structure of vocational interests: The importance of the people – things dimension. Journal of Personality and Social Psychology.

[B37] Gottfredson LS, Brown D (2002). Gottfredson's theory of circumscription, compromise, and self-creation. In Career choice and development.

[B38] Matthews G, Deary IJ, Whiteman MC (2003). Personality traits.

[B39] McCrae RR, Costa PT (2003). Personality in adulthood: A five-factor theory perspective.

[B40] Biggs JB (1987). Student approaches to learning and studying.

[B41] Fox RA, McManus IC, Winder BC (2001). The shortened Study Process Questionnaire: an investigation of its structure and longitudinal stability using confirmatory factor analysis. British Journal of Educational Psychology.

[B42] Bock RD, Jones LV (1968). The measurement and prediction of judgement and choice.

[B43] Borg I, Groenen P (1997). Modern multidimensional scaling: Theory and applications.

[B44] Kruskal JB, Wish M (1978). Multidimensional scaling.

[B45] Arabie P, Carroll JD, DeSarbo WS (1987). Three-way scaling and clustering.

[B46] McManus IC, Richards P (1984). An audit of admission to medical school: 1. Acceptances and rejects. British Medical Journal.

[B47] McManus IC, Richards P (1986). Prospective survey of performance of medical students during preclinical years. British Medical Journal.

[B48] McManus IC, Richards P, Maitlis SL (1989). Prospective study of the disadvantage of people from ethnic minority groups applying to medical schools in the United Kingdom. British Medical Journal.

[B49] McManus IC, Richards P, Winder BC, Sproston KA (1998). Clinical experience, performance in final examinations, and learning style in medical students: prospective study. British Medical Journal.

[B50] McManus IC, Richards P, Winder BC, Sproston KA, Styles V (1995). Medical school applicants from ethnic minorities: identifying if and when they are disadvantaged. British Medical Journal.

[B51] McManus IC, Richards P, Winder BC (1999). Intercalated degrees, learning styles, and career preferences: prospective longitudinal study of UK medical students. British Medical Journal.

[B52] Royal Commission (1968). Royal Commission on Medical Education (The Todd Report), Cmnd 3569.

[B53] Prediger DJ (1982). Dimensions underlying Holland's Hexagon: Missing link between interests and occupations?. Journal of Vocational Behavior.

[B54] McManus IC, Sproston KA (2000). Women in hospital medicine: Glass ceiling, preference, prejudice or cohort effect?. Journal of Epidemiology and Community Health.

[B55] Woodall A, Pickard M (1997). Mature entrants to medicine. BMJ Classified.

[B56] Borges NJ, Osmon WR (2001). Pesonality and medical speciality choice: Technique orientation versus people orientation. Journal of Vocational Behavior.

[B57] Barrett PT, Petrides KV, Eysenck SBG, Eysenck HJ (1998). The Eysenck Personality Questionnaire: An examination of the factorial similarity of P, E, N, and L across 34 countries. Personality and Individual Differences.

[B58] Gale R, Grant J (2001). Sci45: The development of a specialty choice inventory.

[B59] Gale R, Grant J (2002). Sci45: the development of a speciality choice inventory. Medical Education.

