# Supplementary material for: Mapping medical careers: Questionnaire assessment of career preferences in medical school applicants and final-year students
Source: BMC Med Educ. 2004 Oct 1;4:18. doi: 10.1186/1472-6920-4-18 (PMC524180; doi:10.1186/1472-6920-4-18)
Supplement: Additional File 1 — Additional analyses of data [file 1472-6920-4-18-S1.pdf]

Supplementary Material

i) *Sample sizes*. Table S1 (below) shows the number of individuals in the various surveys who had valid career preference data (although not all had answered all of the career preference questions). The numbers in the parentheses are the number of individuals who had complete data on all of the careers, and whose data were used in the INDSCAL analyses. These numbers vary between applicants/entrants and final-year students, and between cohorts, because of differing student knowledge about some more unusual careers (which varied between studies – see main text), and hence not all students felt able to make judgements on all careers.

Table S1:

	Non-mature applicants		Mature applicants		Total
	Male	Female	Male	Female	
**1981 cohort**					
Applicants	589 (276)	387 (172)	104 (56)	55 (34)	1135 (538)
Final-year students	162 (155)	143 (135)	20 (17)	5 (5)	330 (312)
**1986 Cohort**					
Applicants	978 (573)	831 (447)	134 (76)	89 (52)	2032 (1118)
Final-year students	178 (139)	170 (137)	13 (10)	15 (15)	376 (301)
**1991 Cohort**					
Entrants	1340 (733)	1424 (777)	117 (78)	92 (50)	2973 (1638)
Final-year students	564 (564)	750 (750)	70 (70)	53 (53)	1437 (1437)

ii) *Potential response bias*. Two possible forms of response bias need to be considered in our analysis. Firstly, in the 1991 cohort we report the mapping analyses only on the *entrants* to medical school, whereas in the rather smaller 1981 and 1986 cohorts we describe data on *applicants* to medical school, some of whom will have been rejected. Secondly, although our response rates are uniformly high in the studies of applicants/entrants, averaging about 90% across the three studies, they are somewhat lower in our studies of final-year students, averaging about 55% across the studies. It is therefore possible that there are systematic differences in career preferences between respondents and non-respondents in the final-year surveys. We will consider these questions only in the 1991 cohort, since it is by far the largest, and hence the results will be more informative.

We assess response bias by considering data from the 1991 cohort (and it should be noted that this refers to the *entire* dataset of applicants from the 1991 cohort, and not just the entrants). Bias due to differences between entrants (accepted applicants) and rejects can be assessed by comparing career preferences at application in the two groups of students. Bias due to differential response in the final-year students can be partly assessed by comparing career preferences *at application* in entrants who did or did not respond to the final-year questionnaire.

Table S2 shows the mean preference for each of the careers in those accepted for medical school and those who were rejected. There are 27 significance tests and hence an appropriate alpha level after a Bonferroni correction is 0.05/27 or about 0.001. Nine of the careers show significant differences on that basis. However the absolute effect size is small, the largest difference being in Medical Administration, where the scores differ by 1.78 - 1.62 = 0.16, which is one fifth of a scale point, a difference which is also equivalent to 0.20 standard deviations. Although the differences are of some interest, they are few and small in terms of their absolute and relative size. Overall they are unlikely to be biassing the mapping process described in the main paper.

Table S3 compares the preferences at application of those individuals in the 1991 cohort study who did or did not respond to the final-year questionnaire. Once again there are 27 significance tests, and therefore a Bonferroni corrected level of 0.001 was used. None of the probabilities reached that level, the lowest being 0.006. In this case too, the sample size is large, and it can therefore be concluded that there is unlikely to be any bias in the final-year samples due to differential non-response.

Table S2: Interest in different medical careers in the 1991 cohort study for accepted and rejected applicants to medical school. Scores are in the range from 1 (definite intention not to go into the career) to 5 (Definite intention to go into the career). Differences in means were assessed using Student's t-statistic, which is shown in the final column, along with the significance level (which is uncorrected – see text).

Mean, SD, N	*Not accepted at medical school*	*Accepted at medical school*	*t, P*
Anaesthetics	2.43, .845, 2106	2.45, .763, 2690	-1.113, .266
Radiology	2.22, .794, 2097	2.24, .751, 2672	-.954, .340
Ophthalmology	2.55, .883, 1839	2.55, .821, 2293	.047, .963
Dermatology	2.50, .863, 1996	2.46, .791, 2590	1.680, .093
Pathology	2.87, .979, 2062	2.74, .928, 2706	4.812, <.001
Ear, nose and throat surgery	2.90, .907, 2172	2.88, .830, 2753	.995, .320
Surgery	3.56, .980, 2239	3.45, .926, 2852	4.142, <.001
Traumatic and orthopaedic surgery	3.19, .929, 2020	3.18, .910, 2583	.192, .848
Medicine in hospital	3.65, .792, 2203	3.62, .707, 2818	1.291, .197
Obstetrics and Gynaecology	3.13, 1.017, 2129	3.10, .931, 2754	1.139, .255
Paediatrics	3.51, 1.000, 2124	3.48, .904, 2801	1.148, .251
Psychiatry	2.90, 1.116, 2171	2.91, 1.037, 2818	-.170, .865
General Practice (single-handed)	2.76, 1.102, 2237	2.63, 1.021, 2883	4.434, <.001
General Practice (small group)	3.08, 1.100, 2268	3.03, 1.057, 2893	1.498, .134
General Practice (Health centre)	2.95, 1.069, 2226	2.84, 1.019, 2862	3.606, <.001
Basic medical sciences	2.44, .877, 2020	2.44, .867, 2574	.215, .829
Medical research	2.81, 1.105, 2242	2.69, 1.062, 2857	3.785, <.001
Laboratory medicine	2.40, 1.051, 2206	2.27, .971, 2820	4.247, <.001
Pharmaceutical medicine	2.09, .978, 2207	1.92, .881, 2804	6.425, <.001
Medical administration	1.78, .849, 2148	1.62, .743, 2790	6.768, <.001
Public health / Community medicine	2.33, .966, 2145	2.22, .892, 2740	3.982, <.001
Forensic medicine	2.88, 1.075, 2149	2.84, .997, 2790	1.321, .186
Industrial medicine	2.02, .909, 2079	1.96, .822, 2660	2.320, .020
Armed forces	2.08, 1.154, 2210	2.01, 1.111, 2817	2.284, .022
Infectious diseases	3.03, .863, 2141	2.96, .817, 2710	2.701, .007
Genito-urinary medicine	2.51, .845, 1996	2.51, .801, 2525	-.0534, .958
Geriatric medicine	2.35, .914, 2071	2.29, .864, 2741	2.209, .027

Table S3: Interest in different medical careers *at application* in the final-year students in the 1991 cohort study who did or did not return the final-year questionnaire. Scores are in the range from 1 (definite intention not to go into the career) to 5 (Definite intention to go into the career). Differences in means were assessed using Student's t-statistic, which is shown in the final column, along with the significance level (which is uncorrected – see text).

Mean, SD, N	*Did not return final-year questionnaire*	*Did return final- year questionnaire*	*t, P*
Anaesthetics	2.48, .758, 445	2.45, .755, 1269	.759, .448
Radiology	2.22, .784, 440	2.24, .732, 1253	-.520, .603
Ophthalmology	2.59, .802, 376	2.52, .815, 1081	1.470, .142
Dermatology	2.43, .813, 429	2.47, .786, 1238	-1.000, .317
Pathology	2.70, .938, 436	2.77, .908, 1286	-1.280, .201
Ear, nose and throat surgery	2.91, .852, 452	2.87, .831, 1291	.896, .370
Surgery	3.49, .916, 470	3.40, .917, 1329	1.901, .057
Traumatic and orthopaedic surgery	3.18, .897, 408	3.18, .901, 1234	-.114, .909
Medicine in hospital	3.65, .735, 463	3.61, .682, 1331	1.027, .305
Obstetrics and Gynaecology	3.11, .917, 439	3.12, .914, 1310	-.148, .882
Paediatrics	3.46, .947, 461	3.51, .871, 1322	-.960, .337
Psychiatry	2.88, 1.082, 461	2.90, 1.024, 1328	-.314, .753
General Practice (single-handed)	2.68, 1.046, 469	2.63, 1.011, 1355	.994, .320
General Practice (small group)	3.10, 1.059, 472	3.04, 1.065, 1358	.991, .322
General Practice (Health centre)	2.91, 1.041, 463	2.84, 1.011, 1345	1.220, .222
Basic medical sciences	2.36, .880, 428	2.43, .867, 1212	-1.395, .163
Medical research	2.72, 1.077, 466	2.65, 1.062, 1345	1.295, .196
Laboratory medicine	2.18, 1.009, 460	2.28, .958, 1326	-1.864, .062
Pharmaceutical medicine	1.94, .898, 457	1.91, .872, 1321	.682, .495
Medical administration	1.69, .736, 447	1.58, .722, 1321	2.750, .006
Public health / Community medicine	2.24, .883, 448	2.23, .898, 1292	.369, .712
Forensic medicine	2.86, .977, 453	2.86, .988, 1322	-.123, .902
Industrial medicine	1.95, .848, 438	1.95, .816, 1251	-.017, .986
Armed forces	2.03, 1.085, 463	1.99, 1.108, 1323	.537, .591
Infectious diseases	2.96, .836, 442	2.99, .788, 1274	-.657, .511
Genito-urinary medicine	2.50, .782, 406	2.53, .778, 1191	-.739, .460
Geriatric medicine	2.30, .882, 431	2.29, .842, 1309	.336, .737
